# Comparison of Retinal Microvessel Blood Flow Velocities Acquired with Two Different Fields of View

**DOI:** 10.1155/2017/2895982

**Published:** 2017-07-05

**Authors:** Jin Zhou, Min Li, Wan Chen, Ye Yang, Liang Hu, Liang Wang, Hong Jiang, Jianhua Wang

**Affiliations:** ^1^Guangzhou Women and Children's Medical Center, Guangzhou, Guangdong, China; ^2^Bascom Palmer Eye Institute, University of Miami, Miami, FL, USA; ^3^Department of Ophthalmology, Shanghai General Hospital, School of Medicine, Shanghai Jiaotong University, Shanghai, China; ^4^Zhongshan Ophthalmic Centre, Sun Yat-sen University, Guangzhou, Guangdong, China; ^5^School of Optometry and Ophthalmology, Wenzhou Medical University, Wenzhou, China; ^6^Krieger School of Arts and Sciences, Johns Hopkins University, Baltimore, MD, USA; ^7^Department of Neurology, University of Miami, Miami, FL, USA

## Abstract

To compare the different retinal blood flow velocities (BFVs) acquired with different fields of view (FOVs) using the retinal function imager (RFI), twenty eyes of twenty healthy subjects were enrolled in the study. Retinal microvessel BFV in the macula was acquired with both a wide FOV (35 degrees, 7.3 × 7.3 mm^2^) and a commonly used small FOV (20 degrees, 4.3 × 4.3 mm^2^). The 35-degree FOV was trimmed to be equivalent to the 20-degree FOV to compare the BFVs of the similar FOVs using different settings. With the 35-degree FOV, both retinal arteriolar and venular BFVs were significantly greater than the 20-degree FOV (*P* < 0.001). When the 20-degree FOV was compared to the trimmed equivalent 20-degree FOV acquired using the 35-degree FOV, significant BFV differences were found in both the arterioles (*P* = 0.029) and venules (*P* < 0.001). This is the first study to compare retinal blood flow velocities acquired with different FOVs using RFI. The conversion factor from 35 degrees to 20 degrees is 0.95 for arteriolar BFV and 0.92 for venular BFV, which may be used for comparing BFVs acquired with different FOVs.

## 1. Introduction

Retinal blood flow velocities (BFVs) may provide useful information about microcirculation in the retina [1] and possibly reflect the microcirculation in the brain since the microvasculature in the retina and brain is similar anatomically and physiologically [2–4]. Retinal microcirculation has been evaluated by a series of methods including video fluorescein angiography [5], ultrasound flowmetry [6], the blue-field simulation technique [7], scanning laser Doppler flowmetry [8], and intravenous fluorescein angiography (IVFA) [9]. Compared with the abovementioned techniques, the retinal function imager (RFI) provides an in vivo, noninvasive method for direct measurement of the BFVs in retinal vessels without the use of contrast agents [10]. RFI was successfully used to determine the changes in retinal microcirculation in various diseases. In the majority of previous studies, the BFV was measured using the 20-degree FOV [11–17] while other studies used the 35-degree FOV [18–21]. Both settings successfully determined the differences between the diseased eyes and controls. However, the results appeared to vary when the different FOVs were used (Table 1). This discrepancy may prevent direct comparisons among studies and cross references for future studies. The goal of the present study was to compare RFI BFVs acquired with different FOVs.

## 2. Materials and Methods

Twenty healthy subjects, consisting of 10 men and 10 women, with ages ranging from 18 to 50 years (32.5 ± 8.0 years, mean ± standard deviation) were enrolled in the present study. The protocol was approved by the institutional review board of the University of Miami. All subjects signed consent forms. The exclusion criteria included ocular media opacity and any previous ocular surgeries except for a remote history of cataract extraction (at least 6 months prior to enrollment).

The RFI system (RFI 3000, Optical Imaging Ltd., Rehovot, Israel) has been well described previously [10, 14]. Briefly, RFI is a fundus camera adapted with an advanced digital camera, which captures a series of 8 images at 60 frames/second. During imaging, the stroboscopic illumination enables the device to capture the movement of red blood cells. Since hemoglobin is a naturally high-contrast chromophore, the RFI uses the reflectance contrast from the series of eight images to track blood flow. The software then automatically calculates BFV in retinal vessels from the second and tertiary branches [22]. To control the effect of pulsation on the measurement, image acquisition is synchronized with the given period of the subject's cardiac cycle through a probe that is attached onto the fingertip or earlobe.

**Figure 1:**
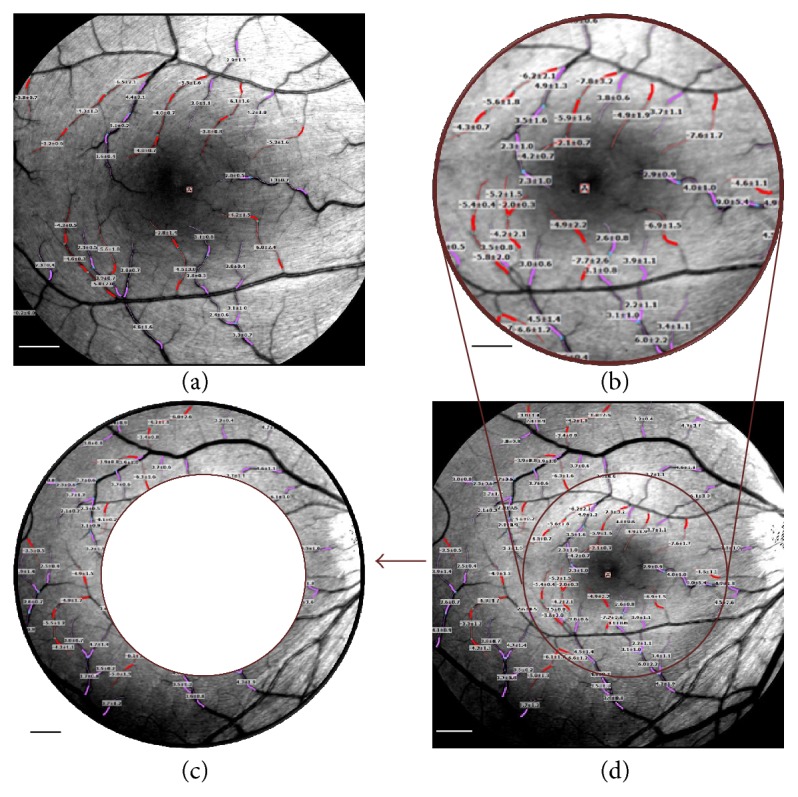
Comparisons of retinal microvessel blood flow velocity (BFV) measured using RFI with different fields of view (FOVs). Retinal microvessel BFVs centered on the fovea were acquired with the small FOV ((a) 20 degrees, 4.3 × 4.3 mm^2^) and the wide FOV ((d) 35 degrees, 7.3 × 7.3 mm^2^). The 35-degree FOV was divided into the inner field (b), equivalent to the 20-degree FOV, and the outer field (c); the peripheral region located beyond the 20-degree FOV BFVs (expressed in mean ± standard deviation) of the secondary and tertiary branches were measured. Arterioles are marked in pink and venules are marked in red. Minus velocity indicates a flow toward the tissue and therefore the vessel is an arteriole. Bars = 500 *μ*m.

By the same experienced photographer, one eye of each subject was imaged using RFI. Three or more well-focused sessions with at least four good images per session were obtained for each eye. The 35-degree FOVs were acquired first then followed by the 20-degree FOVs. To obtain BFV, the vessels were marked manually in the RFI software and the arteriolar and venular BFVs were calculated by the software. The two FOVs were captured by using the camera settings of 20 and 35 degrees. The FOV of the 20 degrees is 4.3 × 4.3 mm^2^ and the FOV of the 35 degrees is 7.3 × 7.3 mm^2^. The same grader performed segmentation of the retinal vasculature using the RFI software in two independent sessions with segments of 60–90 pixels with the 35° FOVs and 100–150 pixels with the 20° FOVs per manufacturer's recommendation. The 35-degree FOV was divided into the inner field, equivalent to the 20-degree FOV, and the outer field, the peripheral region located beyond the 20-degree FOV (Figure 1). After velocities of all measured vessel segments in the 35-degree FOVs were obtained, the fundus images and measurement results were exported. To outline the trimmed 20-degree FOV, the image obtained with the 20-degree setting was rescaled into the equivalent area by shrinking the dimension from 1024 × 1204 pixels to 603 × 603 pixels (factor = 4.3 mm in 20-degree FOV/7.3 mm in 35-degree FOV). By aligning with the retinal vessels, the two images were laid and a circle of 2.15 mm was drawn to separate the inner and outer regions according to the laid 20-degree FOV image. Using the vessel identifications shown on the 35-degree FOVs, all results of BFVs were read off in the exported data for the inner and outer regions.

Statistical analysis was performed with a statistics package (SPSS, ver. 16.0, IBM, Armonk, NY). Repeated measures analysis of variance (Re-ANOVA) was used to test the variation, and post hoc tests were used to test pair-wise differences of BFVs between FOVs and zones. Pearson's regression was used to determine the relation of BFVs between the inner and outer zones obtained using 35-degree FOV and between 20 and 35 degrees of FOVs. The Bland-Altman plot was used to determine the 95% limit of agreement of BFVs between 20-degree FOV and the equivalent 20-degree FOV trimmed from the 35-degree FOV. The 95% limit of agreement (LoA) was calculated as 1.96× the standard deviation of the difference between the two measurements. Statistical significance was determined by a 2-tailed *P* value of 0.05 (*P* < 0.05). The sample size was determined using a software program (Gpower, Ver. 3.0) developed by Faul et al. [23]. To detect the 0.20 differences between FOVs in both arterioles and venules, a sample size of 15 subjects would be enough with a detection power of 0.8.

## 3. Results

**Figure 2:**
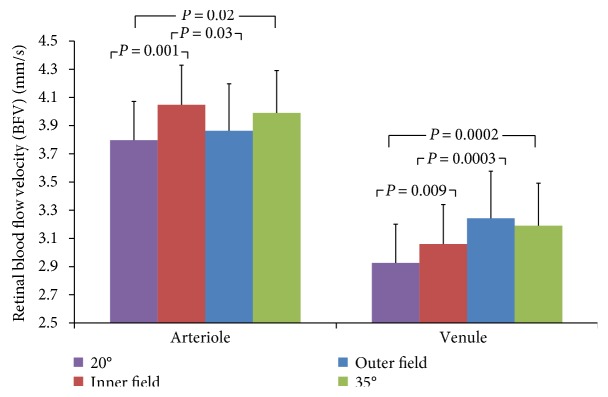
Comparison of retinal BFVs with different FOVs. The average BFVs in the retinal arterioles and venules were measured with the RFI device. The average BFVs of the 20-degree FOV were significantly lower than those of the 35-degree FOV and the inner field, which was equivalent to the 20-degree FOV in both the arterioles and venules (*P* < 0.05). The average BFV of arterioles was significantly higher in the inner region than the arteriolar BFV in the outer region, which was the peripheral region that is not included in the corresponding 20-degree FOV (*P* = 0.03). However, the average venular BFV of the inner field was significantly lower than the outer field venular BFV (*P* = 0.003). Compared to the equivalent 20-degree FOV trimmed from the 35-degree FOV, the average BFVs of the 20-degree FOV acquired with the 20-degree FOV setting were significantly different in both arterioles and venules (*P* < 0.05, Figure 2). Bars = standard error.

Approximately 10% of the vessels were rejected in both 20- and 35-degree FOVs for both arterioles and venules because the relative standard deviation of the BFVs was larger than 0.45 [10, 13], recommended by the manufacturer (Table 2). Although small changes in image centering may possibly induce more the peripherally located vessel segments to be excluded, no such evidence was found. With the 35-degree FOV, the average retinal arteriolar BFV was 4.0 ± 0.4 mm/s (mean ± SD), which was significantly greater than that acquired with the 20-degree FOV (3.8 ± 0.4 mm/s, *P* = 0.02, Table 2 and Figure 1). The average venular BFV with the 35-degree FOV was 3.2 ± 0.3 mm/s, which was also greater than that acquired with the 20-degree FOV (2.9 ± 0.3, *P* = 0.0002). The average arteriolar BFV of the inner field equivalent to 20-degree FOV (4.1 ± 0.4 mm/s) was significantly greater than that of the outer field (3.9 ± 0.5 mm/s, *P* = 0.03). However, the inner venular average BFV (3.1 ± 0.3 mm/s) was significantly lower than the outer average venular BFV (3.2 ± 0.3 mm/s, *P* = 0.003, Figure 2). Compared to those of the equivalent 20-degree FOV trimmed from the 35-degree FOV, the BFVs of the 20-degree FOV acquired with the 20-degree FOV setting were significantly different in both arterioles and venules (*P* < 0.05, Figure 2). In addition to having a lower arterial BFV, the outer field also had fewer arteriolar segments (12 ± 7) than the inner field (21 ± 5) (*P* < 0.001). However, there was no difference in the number of venular segments between the inner (20 ± 5) and outer fields (17 ± 5, *P* = 0.11). The conversion factor from 35 degrees to 20 degrees is 0.95 ± 0.06 for arteriolar BFV and 0.92 ± 0.07 for venular BFV, which may be used for comparing BFVs acquired with different FOVs.

**Figure 3:**
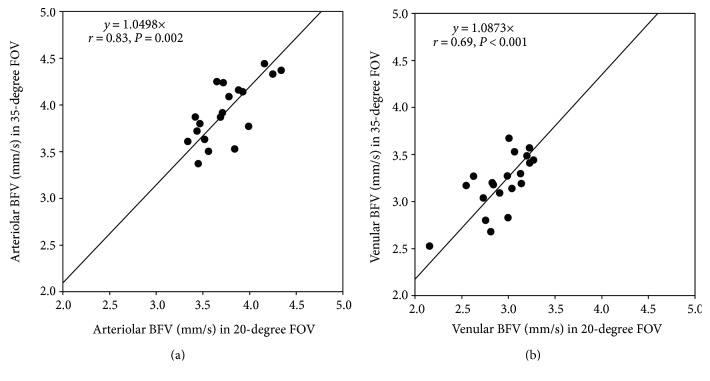
Relationship between the BFVs acquired with the 20- and the 35-degree FOVs. Correlations were found between the BFVs of the 20- and 35-degree FOVs. Conversion factors ((a) arteriole, (b) venule) were extracted from the respective linear expressions, which allow the BFV to be converted between the FOVs.

**Figure 4:**
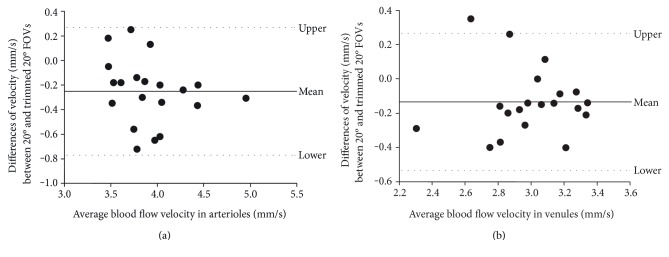
Bland-Altman plot of the difference between the BFVs acquired with the 20-degree FOV and trimmed inner field (region equivalent to 20-degree FOV). Bland-Altman method was used to assess the limit of agreement of the BFVs acquired with the 20-degree FOV and trimmed inner field in arterioles (a) and venules (b). Note that the solid and dashed lines indicate the mean difference and 95% limit of agreement.

**Figure 5:**
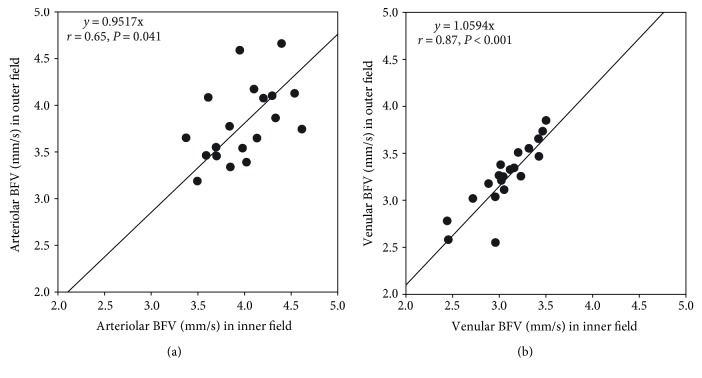
Relationship between the blood flow velocities (BFVs) of the inner and outer fields. There were significant correlations of the BFV between the inner and outer fields in the arterioles (a) (*r* = 0.65, *P* = 0.041) and venules (b) (*r* = 0.87, *P* < 0.001).

Correlations were found between the BFVs of the 20- and 35-degree FOVs (arteriole: *r* = 0.83, *P* = 0.002; venule: *r* = 0.69, *P* < 0.001, Figure 3). The LoA was 0.51 mm/s for arteriolar BFV and 0.38 mm/s for venular BFV between the 20-degree FOV and inner field (i.e., trimmed equivalent 20-degree FOV, Figure 4). Furthermore, there were significant correlations of the BFV between the inner and outer fields in the arterioles (A, *r* = 0.65, *P* = 0.041, Figure 5) and venules (B, *r* = 0.87, *P* < 0.001, Figure 5).

## 4. Discussion

Previous studies had employed different FOV settings for measuring BFV. Of the 12 available previously published studies that measured the BFV of healthy eyes with the RFI, 9 studies used the 20-degree FOV [1, 4, 11–14, 16, 17, 24], which yielded arteriolar BFVs ranging from 3.15 to 4.45 mm/s and venular BFVs ranging from 2.50 to 3.20 mm/s. On the other hand, 4 studies used the 35-degree FOV [18–21], which yielded arteriolar BFVs ranging from 3.16 to 4.70 mm/s and venular BFVs ranging from 3.15 to 3.20 mm/s. Although BFVs obtained with 35-degree FOV are apparently higher than those obtained with the 20-degree FOV, it is impossible to compare the BFVs among these studies since they were acquired with different FOV settings and eyes. The present study is the first study to use both the 20- and 35-degree FOVs on the same eyes simultaneously and compare the resulting BFVs. Our results showed that different FOVs yielded different BFVs, which could not be directly compared. Caution should be taken when comparing BFVs among studies. One possible solution would be applying a conversion factor which converts the BFVs between the 20- and 35-degree FOVs. Based on our studies, the conversion factor from 35 degrees to 20 degrees is 0.95 for arteriolar BFV and 0.92 for venular BFV.

The BFV differences between the different FOVs could be explained by the characteristic layout of the retinal vasculature in the macula, which has a unique vascular network that allows the retina, including the avascular zone (i.e., the fovea), to be sufficiently supplied. As extensions of the central retinal artery, these retinal vessels are responsible for the total ocular blood flow to the inner retinal layers. The special layout of these vessels ensures adequate perfusion to these intraretinal layers, especially the fovea, which has no capillaries. Normally, 20–24 alternating arteriolar and venular branches are densely surrounding and terminating at the fovea. The 20-degree FOV contains the vessels between the superior and inferior temporal arch of the retinal arteries, while the outer field has more venules originated from the intravenous bow. In addition, while the 20-degree FOV contains both secondary and tertiary branches, the area outside the 20-degree FOV predominantly contains secondary branches since the area mostly includes the main arteriole. Furthermore, a 35-degree FOV is 7.3 × 7.3 mm^2^ in a 35-degree setting, while a 20-degree FOV is 4.3 × 4.3 mm^2^ with the 20-degree setting. The 35-degree FOV covers a ~3 times larger area than the 20-degree FOV. Due to the special arrangement of the vessel distribution, when centered on the fovea, the 35-degree FOV covers a larger area, which will likely include more secondary branches with faster velocities. This vessel distribution may result in a higher average velocity than that of the 20-degree FOV. Therefore, the retinal vascular layout could explain the results of the present study. This viewpoint is further supported by the results between the inner and outer fields.

Furthermore, the focusing process appears to be easier during imaging with the 35-degree FOV since the 35-degree FOV contains more vessels and has a deeper focal depth than that of the 20-degree FOV. Thus, the 35-degree FOV may be advantageous in clinical applications and more reliable for measuring BFV. It may also be sensitive to changes in the retinal microcirculation. On the other hand, the 20-degree FOV has higher lateral resolution and the tertiary branches of the vessels are better visualized, which may offset the drawback mentioned above and theoretically improve the precision and accuracy of the measurement. The present work did not compare the repeatabilities of the different fields of view. Further studies are needed to determine which FOV provides more precise BFV measurements.

The good relations between the BFVs in the 20-degree and 35-degree FOVs were expected and the interpretation should be with great caution. The vessels measured in the 20-degree FOVs appeared to contribute more than 50% of the vessels measured in the 35-degree FOVs. Therefore, a strong relation must be expected, which appears to reflect the relative distribution of the vessels between the inner and outer fields.

The BFV difference between the 20-degree FOV and the trimmed equivalent 35-degree FOV was unexpected. This may be a systematic error that occurred due to the limited area of the 20-degree FOV. The RFI acquires multiple sessions and registers them by aligning the vessels. Because the FOVs of each imaging session did not always match, the vessel segments around the edge of the image were often less than 100 pixels. In accordance with the manufacturer's suggestions, the vessel length needs to be more than 100 pixels for accurate processing [17]. Therefore, some of the vessel segments were excluded, which makes the FOV even smaller than the trimmed equivalent 35-degree FOV, resulting in the possible systematic error. It may be also possible that the systematic difference is due to slight calibration inaccuracies. The trimmed area was based on the difference of the FOVs between the 20- and 35-degree FOVs. If the prespecified micron-per-pixel calibration has a slight error, the systematic difference between the 20-degree FOV and the trimmed 20-degree FOV may be induced. One additional possibility would be the true variation in BFV systematically. For example, the 35-degree FOVs were acquired and followed by the 20-degree FOVs. Klefter et al. reported the short-term variability with the LoA up to 1.1 mm/s [16]. Further studies are needed to test the repeatabilities with different FOVs in different imaging orders which may validate the viewpoint. It would be worth noting that the difference between the 20-degree FOVs and trimmed 20-degree FOVs was only ~3–5%, which is relatively small compared to the changes by hyperoxia (~15% in both arterioles and venules) [16] and in diseased conditions such as multiple sclerosis (~20%) [4].

The present study has several limitations. First, even though significant BFV differences between the different FOVs were found, the sample size may be a concern. There were 20 subjects in the present study and most likely the differences between FOVs are true differences as indicated by statistical analysis. Nevertheless, further studies with larger sample sizes may be needed to further confirm the BFVs of the different FOVs. Second, some vessel segments were rejected if the standard deviation of the BFV was larger than 0.45, recommended by the manufacturer [10, 13]. Consequently, there were not exactly the same vessel segments for each eye included in the 20-degree FOVs and the trimmed 20-degree FOVs for comparison. This may also contribute to the discrepancy of the BFV measurements in between. Third, no diseased eyes were included in the present study. Further confirmation with diseased eyes is necessary because this BFV difference between the different FOVs may also exist in the diseased eye. Third, as the only available method in the RFI system, the semiautomated BFV acquisition method was used in the present study, which may lead to measurement errors. The RFI manufacturer is currently developing automated BFV acquisition software, which may prevent these errors. Lastly, it is worth noting that different velocities were found using the same FOVs in different studies [1, 4, 11–21, 24], which may be due to the different human subjects, imaging qualities, and numbers of vessel segments. These potential discrepancies were not addressed in the present study.

In summary, this is the first study to compare retinal blood flow velocities acquired with different FOVs using RFI. Retinal blood flow velocities were significantly different when different FOVs were used. Conversion factors may be used for comparing BFVs acquired with different FOVs.

## Figures and Tables

**Table 1:** Overview of RFI blood flow velocity analysis as reported in clinical studies.

Authors	FOV (20°/35°)	*N* (eyes)	Arteriolar velocity (mm/s)	Venular velocity (mm/s)
Present work	20°	20	3.8 ± 0.4	2.9 ± 0.3
Burgansky-Eliash et al. [14]	20°	51	4.19 ± 0.99	3.03 ± 0.59
Beutelspacher et al. [11]	20°	5	4.3 (3.7–4.8)*^ψ^*	3.0 (2.7–3.3)*^ψ^*
Burgansky-Eliash et al. [1]	20°	51	4.1 ± 0.9	2.9 ± 0.5
Burgansky-Eliash et al. [13]	20°	114	4.2 ± 0.9	3.3 ± 0.8
Klefter et al. [16]	20°	16	4.0 ± 0.9	3.2 ± 0.7
Burgansky-Eliash et al. [12]	20°	53	4.3	2.9
Somfai et al. [17]	20°	10	4.45 ± 0.76	3.17 ± 0.84
Burgansky-Eliash et al. [24]	20°	51	4.2 (3.9–4.6)	3.0 (2.7–3.3)
Jiang et al. [4]	20°	17	4.10 ± 0.87	3.22 ± 0.89

Present work	35°	20	4.0 ± 0.4	3.2 ± 0.3
Feng et al. [20]	35°^∗^	51	3.93 (3.35, 4.65)*^ψ^*	2.82 (2.39, 3.53)*^ψ^*
Landa et al. [21]	35°	30^∗∗^	4.7 ± 0.6	3.7 ± 0.4
Beutelspacher et al. [18]	35°	12	4.24 ± 1.04	3.33 ± 0.76
Chhablani et al. [19]	35°	18	3.16	3.15

^∗^Information obtained through personal communication. ^∗∗^Data obtained through personal communication. ^*ψ*^Data range in parentheses.

**Table 2:** Retinal blood flow velocity using 20- and 35-degree FOVs.

		Arteriole	Venule
Velocity (mm/s)	20 degrees	3.80 ± 0.37	2.93 ± 0.27
	Inner field	4.05 ± 0.43	3.06 ± 0.28
	Outer field	3.86 ± 0.47	3.24 ± 0.33
	35 degrees	3.99 ± 0.42	3.19 ± 0.30

Velocity ratio	20 degrees/inner field	0.95 ± 0.06	0.96 ± 0.07
	20 degrees/35 degrees	0.95 ± 0.06	0.92 ± 0.07
	Inner/outer	1.05 ± 0.10	0.95 ± 0.06

Vessel segments	20 degrees	20 ± 4	19 ± 2
	Inner field	21 ± 5	20 ± 5
	Outer field	12 ± 7	17 ± 5
	35 degrees	32 ± 10	37 ± 7

Vessel rejection rate (%)	20 degree	8 ± 4	6 ± 4
	35 degrees	10 ± 4	11 ± 4
